# Furcation Involvement Classification: A Comprehensive Review and a New System Proposal

**DOI:** 10.3390/dj6030034

**Published:** 2018-07-23

**Authors:** Andrea Pilloni, Mariana A. Rojas

**Affiliations:** Section of Periodontology, Department of Oral and Maxillo-Facial Sciences, “Sapienza” University of Rome, Rome 00161, Italy; andrea.pilloni@uniroma1.it

**Keywords:** classification, furcation lesions, gingival recession, prognosis

## Abstract

Various classification systems have been proposed to describe furcation lesions and Glickman’s classification for many years seems to have been the most widely utilized in the sole clinical diagnosis with no reference to the prognostic value of the lesion itself. This article reviews the previous classification systems and proposes a new method to classify furcation lesions based on the position of the gingival margin and its relationship with the furcation area (clinically exposed/non-exposed furcation area) providing significant aid for a better understanding of furcation involvements and increases the prognostic value of treatments in the long term.

## 1. Introduction

According to the glossary of terms of the American Academy of Periodontology, a furcation involvement exists when periodontal disease has caused resorption of bone into the bi- or trifurcation area of a multi-rooted tooth [1].

Currently, the proposed classifications are based on the extension of the defect and the degree of horizontal/vertical attachment loss. Glickman in 1953 [2], developed a classification system in order to describe the extension and main characteristics of the furcation defect (Grade I–IV). Hamp, Nyman and Lindhe [3] and Tarnow and Fletcher [4] proposed to measure the horizontal/vertical attachment loss, respectively.

Moreover, other classifications have been proposed in an attempt to describe the anatomy of the furcation more completely, describing the number of remaining bony walls [5], the morphology of the existing bone [6] and the relationship between root trunk and vertical/horizontal attachment loss [7].

Most of the classifications of furcation lesions are unable to convey all the relevant information related to the marginal tissue position and its relationship with the furcation involvement (clinically exposed/non-exposed furcation). This information could be important for diagnosis, prognosis and treatment planning as well as for the communication between clinicians and researchers. Furthermore, with a broad variety of cases with different clinical presentations, it is not always easy to classify all furcation defects according to classification systems in use to date.

The aim of the present review is to summarize the previous classifications that have been proposed in the past years and introduce a new system that provides information on a parameter that has not been taken into account; the gingival marginal tissue position and its relationship with the clinical exposure of the furcation involvement. The goal is to promote this new classification as a promising system for a better understanding of furcation involvements and to predict the prognosis by selecting the correct treatment for each case.

## 2. Classification of Furcation Lesions

Several systems have been proposed based either on the extent of horizontal probing depth into the furcation defect or on the vertical extent of the loss of alveolar bone within the defect (Table 1).

All the classifications proposed to date are showed in Table 1. However, it is important to highlight the main systems used and their limitations.

One of the first proposed classifications was the one by Glickman [2]. This classification system probably is the most widely used and it describes the main characteristics of furcation lesions:

Grade I involvement: it is the incipient or early lesion. The pocket is supra-bony, involving the soft tissue; there is slight bone loss in the furcation area. Radiographic change is not usual, as bone changes are minimal.

Grade II involvement: the bone is destroyed on one or more aspects of the furcation, but a portion of the alveolar bone and periodontal ligament remain intact, thus allowing only partial penetration of the probe into the furcation area. The radiograph may or may not reveal the grade II furcation involvement.

Grade III involvement: the inter-radicular bone is completely absent, but the facial and/or lingual orifices of the furcation are occluded by gingival tissue. Therefore, the furcation opening cannot be seen clinically, but it is essentially a through and through tunnel. If the radiograph of the mandibular molars is taken with a proper angle and the roots are divergent, these lesions will appear on the radiograph as a radiolucent area between the roots. The maxillary molars present a diagnostic difficulty owing to roots overlapping each other.

Grade IV involvement: the inter-radicular bone underneath the roof of furcation is completely destroyed. The gingival tissue is also receded apically so that the furcation opening is clinically visible. The radiographic image is essentially the same as in grade III lesions.

In 1975 Hamp, Nyman and Lindhe [3] proposed a classification system referring to the horizontal attachment loss and based on three degrees:

Degree I: horizontal attachment loss < 3 mm of the total width of the furcation area.
Degree II: horizontal attachment loss > 3 mm but not encompassing the total width of the furcation area.
Degree III: “through and through” destruction of the periodontal tissue in the furcation area.

Ramfjord and Ash [11] proposed the same system but the assigned value is 2 mm instead of 3 mm. Other authors defined as degree I a furcation with an estimated horizontal attachment loss of up to 1/3 and as degree II with more than 1/3 of the tooth diameter, while the class III definition was left unchanged [19].

Later on, a sub-classification referring to the vertical bone loss from the furcation fornix [4] was introduced to complement the horizontal classification (I–III):

Subclass A: vertical bone loss 3 mm or less.
Subclass B: vertical bone loss from 4 to 6 mm.
Subclass C: bone loss from the fornix of 7 mm or more.

### Limitations

Although Glickman’s classification [2] has been extensively used, there are limitations that need be considered:

- The reference point for the classification is the horizontal attachment loss. However, the subjectivity in the term “early or incipient lesion” (grade I) and the absence of the precise numerical values to identify the horizontal attachment loss create difficulties in the classification between grade I and II.
- In Glickman’s grade III and IV furcation lesion the inter-radicular bone is completely absent, with the difference that in grade IV the furcation entrance is exposed. In this case, grade III and IV would represent a single group considering that the reference point of this classification is the horizontal attachment loss and it is the same for both groups. The difference between them could be represented by creating two subgroups.
- In grade I and II furcation lesions the relationship with gingival margin (clinical exposition of the furcation) is not considered. In such cases, a furcation lesion with incipient bone loss (Glickman’s grade I) but clinically exposed for the presence of gingival recession cannot be classified as grade I but neither as grade IV. The same problem arises for Glickman’s grade II.

The classification systems that quantify the horizontal/vertical attachment loss [3,4,11,19] also present limitations that should be considered:

- The classification systems quantifying the horizontal attachment loss give rise to the same problem: in none of them one can clearly differentiate between grade I and II since both use the same reference point (less or greater than one third—less or greater than two or three millimeters) [3,11,19], which means that it is not clear in which group furcation involvements with measures of one 1/3 or 2–3 mm will be included.
- When the furcation lesion is partially or not clinically exposed it is difficult to measure the vertical bone loss [4,7,15,16]. In these cases, a routine radiographic image could help only in cases where the inter-radicular bone loss is completely absent (grade III Glickman), and this will be in turn more precise in lower molars, because the overlapping of structures in upper molars makes diagnosis difficult.

Considering the above limitations and the fact that none of the proposed classification systems to date consider the clinical exposition of the furcation area as a reference point, a more detailed and informative new classification system is proposed. This classification is based on criteria derived from previously mentioned systems [2,3,11,19] incorporating the relationship between furcation horizontal attachment loss and presence/absence of gingival recession (clinically exposed/non-exposed furcation lesion). For this new system the same parameter as in the Carnevale et al. classification [19]—horizontal attachment loss was used; however, instead of using thirds, the measurements are expressed in millimeters as described Hamp et al. in their classification [3], with the difference that different numerical values have been assigned to grade I and II to avoid confusion when there is a furcation lesion with horizontal attachment loss of 3 mm.

## 3. Proposed Classification System for Furcation Lesions

Furcation lesions are divided into two main groups according to the following criteria (Table 2).

**Figure 1:**
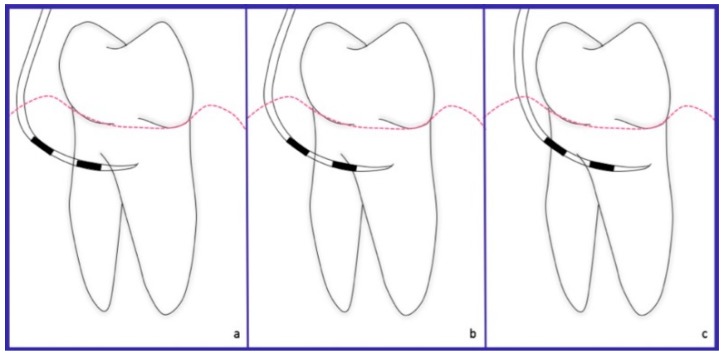
Schematic representation. Non-exposed furcation lesion (NE): (**a**) Class I: Incipient lesion. Horizontal attachment loss of 2 mm or less; (**b**) Class II: Horizontal attachment loss of 3 mm or more; (**c**) Class III: Total horizontal attachment loss (through and through).

**Figure 2:**
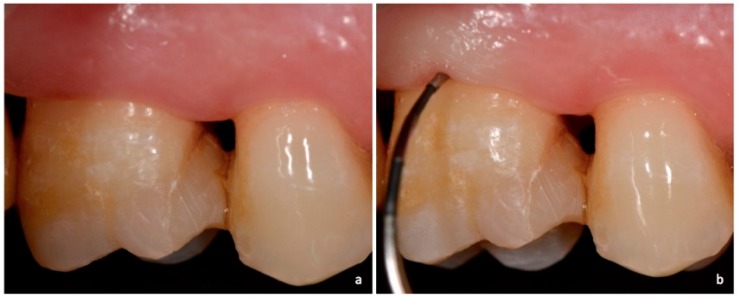
Non-exposed furcation lesion—Class I (NEI): (**a**) First maxillary molar; (**b**) Buccal furcation lesion. Horizontal attachment loss of 2 mm.

**Figure 3:**
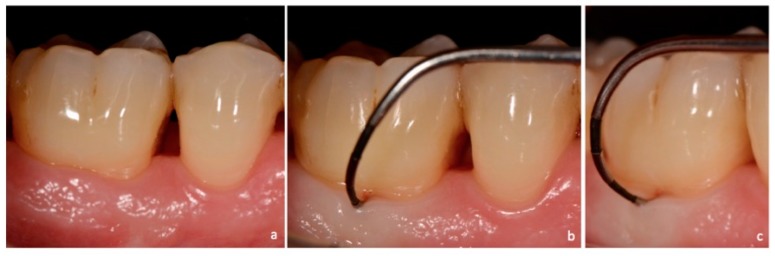
Non-exposed furcation lesion—Class I (NEI): (**a**) First mandibular molar; (**b**)Buccal furcation lesion. Horizontal attachment loss of 2 mm (frontal view); (**c**) Buccal furcation lesion. Horizontal attachment loss of 2 mm (lateral view).

**Figure 4:**
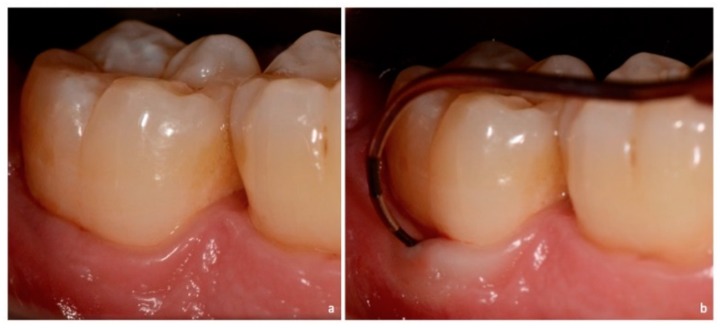
Non-exposed furcation lesion—Class II (NEII): (**a**) Second mandibular molar; (**b**) Buccal furcation lesion. Horizontal attachment loss of 4 mm.

**Figure 5:**
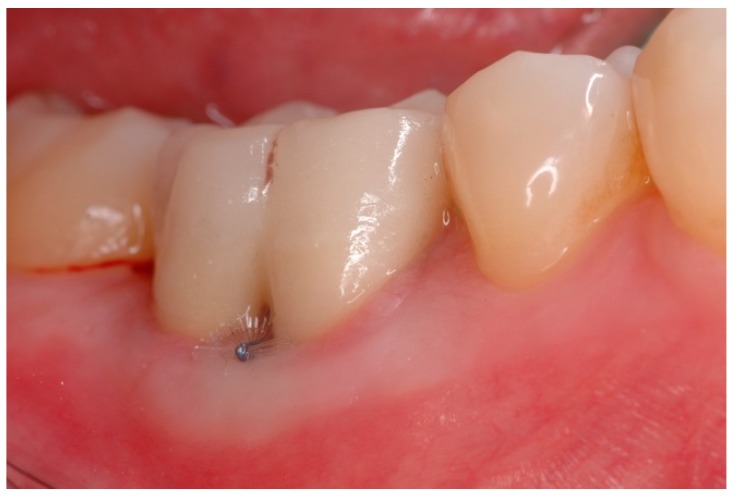
Non-exposed furcation lesion—Class III (NEIII): First mandibular molar. Total horizontal attachment loss (through and through).

Non-exposed (NE): Clinically non-exposed furcation lesion (Figure 1, Figure 2, Figure 3, Figure 4 and Figure 5).

**Figure 6:**
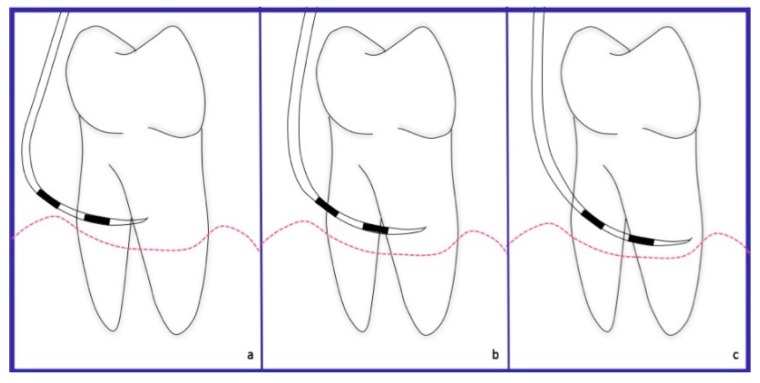
Schematic representation. Exposed furcation lesion (E): (**a**) Class I: Incipient lesion. Horizontal attachment loss of 2 mm or less; (**b**) Class II: Horizontal attachment loss of 3 mm or more; (**c**) Class III: Total horizontal attachment loss (through and through).

**Figure 7:**
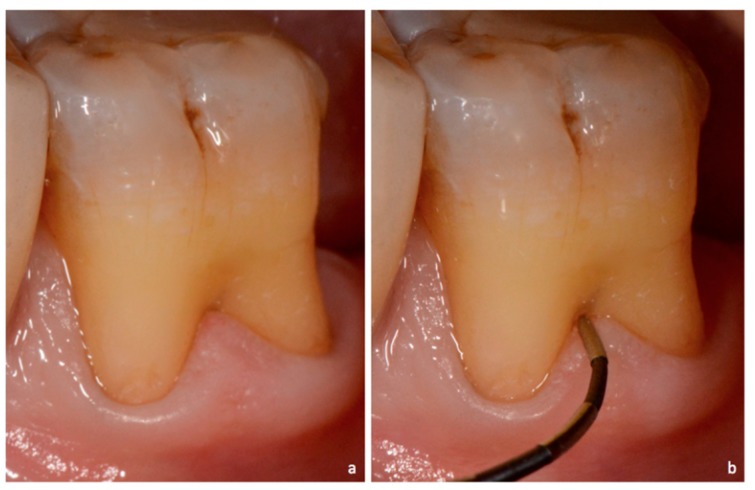
Exposed furcation lesion—Class I (EI): (**a**) First mandibular molar; (**b**) Buccal furcation lesion with horizontal attachment loss of 1 mm.

**Figure 8:**
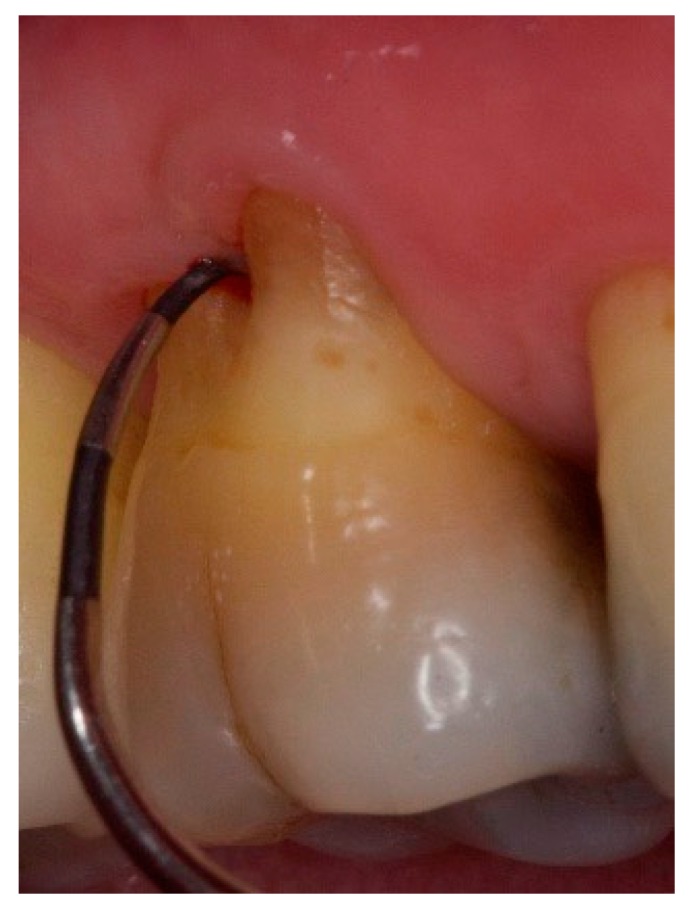
Exposed furcation lesion—Class II (EII): First maxillary molar. Buccal furcation lesion with horizontal attachment loss of 4 mm.

**Figure 9:**
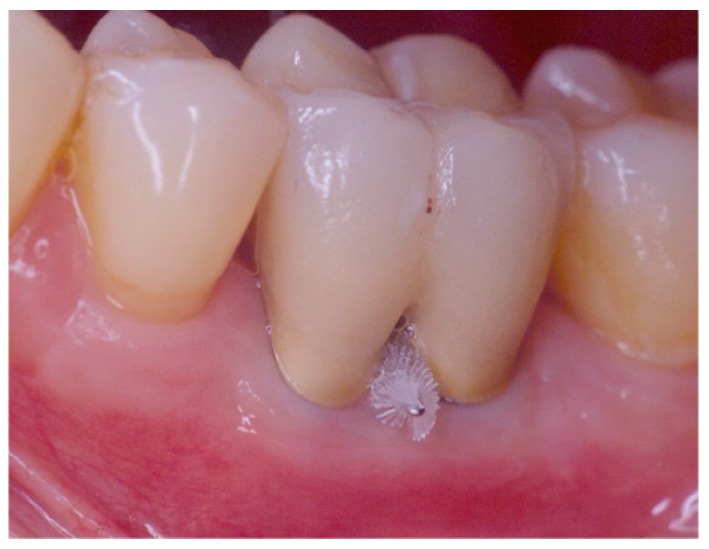
Exposed furcation lesion—Class III (EIII): First mandibular molar. Total horizontal attachment loss (through and through).

Exposed (E): Clinically exposed furcation lesion (Figure 6, Figure 7, Figure 8 and Figure 9).

Considering the main characteristics of the furcation involvement (according to the aforementioned classification systems) [2,3,8,9] each group is further divided in 3 different subgroups:

- Class I: incipient lesion. There is slightly horizontal attachment loss in the furcation area. The examiner probe penetrates two millimeters or less from the entrance of furcation (Figure 2, Figure 3 and Figure 7).
- Class II: partial horizontal bone loss. The examiner probe penetrates three millimeters or more from the entrance of furcation, but there is not a total attachment loss with a through and through opening of the furcation (Figure 4 and Figure 8).
- Class III: total horizontal attachment loss with a through and through opening of the furcation. The inter-radicular bone is completely absent (Figure 5 and Figure 9).

### Marking Guidelines

If a molar presents marginal tissue recession but the furcation is not clinically exposed, it will be considered as non-exposed group (NE) because the criteria for this classification system is the exposure of the lesion more than the presence of gingival recession. For example, if a molar presents a long root trunk, the furcation lesion may not be visible even in the presence of gingival recession.

In order to avoid mistakes in the diagnosis of class II and III lesions—mainly when they are NE—it is important to use two periodontal probes, each of which must be introduced from buccal and lingual (lower molars) and buccal/mesio-palatine–disto-buccal (upper molars); and if they get in contact the diagnosis of a Class III furcation involvement can be confirmed, since many times the anatomical characteristics of the furcation area does not allow to go from “side to side” and this could generate an incorrect diagnosis.

## 4. Discussion

The furcation is an area of complex anatomic morphology that may be difficult or impossible to debride by means of routine periodontal instrumentation [22,23,24]. The treatment of a multi-rooted tooth with a furcation involvement is still a challenge and a problem that has, to date, not been solved. Tooth type and degree of furcation involvement were defined as the most important factors influencing this decision [25].

Therefore, diagnosis and its correct interpretation are essential when establishing an adequate treatment [26].

The aim of this new classification system is to help clinicians to classify those cases, which cannot be categorized into a particular class with any of the current classification. The limitations of the present classification systems can lead to inadequate diagnosis, prognosis and, therefore, treatment planning.

### 4.1. Diagnosis

As discussed in a previous article [26,27], a constant source for measurement error of the furcation lesion is the coronal position of the gingiva relative to the furcation entrance which prevents the desired control of the probe localization.

Zappa et al. [28], compared the classification systems described by Ramfjord and Ash [11] and Hamp et al. [3] with the true depth of the inter-radicular bony defect of molars as assessed by intraoperative measurement. Interestingly, forty-three percent of surgical degree 3 involvements were not recognized when using the Ramfjord index, and 27% when using the Hamp Index. In addition, clinical situations surgically detected as degree 3 furcation lesions were clinically diagnosed as degrees 2, 1, and 0. Therefore, the authors suggested to evaluate factors that could increase the validity of furcation diagnosis.

Considering previous studies [26,27,28] and the fact that clinical furcation diagnoses are used for periodontal treatment planning (and therapy is often chosen according to the clinically detected severity of the involvement) [19], the clinical exposition of the furcation would be an important parameter to be evaluated in the classification of such lesion since a more accurate and reliable diagnosis can be obtained when the furcation entrance is visible (E-clinically exposed furcation) and the possibilities of the under or overestimations can be reduced.

### 4.2. Prognosis and Treatment

Longitudinal prospective [29,30] and retrospective [31,32,33,34,35] studies showed that periodontal treatment shows better results in single-rooted teeth or non-furcated molars with respect to molars with furcation lesions. However, those studies have concluded that the sole presence of a furcation lesion is not enough to assign a questionable or hopeless prognosis to these teeth. McGuire and Nunn [36] evaluated the effectiveness of clinical parameters to develop an accurate prognosis and concluded that, the grade of furcation involvement, determines significantly the prognosis since at completion of periodontal treatment and during maintenance therapy, molars with Class I furcation were lost in 10% of the cases at 12 years, while at 10 years molars with Class II and Class III were lost in 25% and 40% of the cases, respectively. This data allows us to understand- according to this study and the classification used by the authors—how important it is to reduce from a class II to a class I furcation involvement in order to improve the long-term prognosis but, in accordance with the present work: would it be the same with the new proposed classification system? Could the clinical exposition of the lesion (E group) become a critical factor to improve the prognosis? It is important to clarify that this aspect could be one of several ones to be consider when the prognosis of molar with furcation involvement is evaluated. Another important factor is the mobility: longitudinal clinical studies have concluded that mobile teeth with a furcation involvement present a greater risk for attachment loss when compared to teeth without mobility [35,37].

Moreover, according to the new classification of periodontal diseases, once both staging and grading of the periodontitis are established based on evidence of progression, the prognosis can be modified based on the presence of risk factors such as diabetes and smoking. Therefore, these factors could also modify the prognosis of furcated teeth [38].

On the other hand, previous studies comparing scaling and root planing in furcation area with and without surgical access [22,23], have found better results when the treatment was performed with surgical access; although, as well as longitudinal studies [3,31], they concluded that the multi-rooted tooth with furcation lesions could be maintained with adequate periodontal support therapy. Therefore, if we consider the fact that scaling and root planing in the furcation area is more effective with surgical access [22,23], we could infer that it would be more effective also when the furcation lesion is clinically visible (E-clinically exposed furcation lesion).

In fact, a recent retrospective study [39] concluded that, the lower survival rate observed in molars with a furcation involvement (FI) Grade II/III after active periodontal therapy [40] is based on the fact that the removal of the biofilm in this type of FI cannot be performed by the patient without modifying the anatomy of the inter-radicular area, i.e., non-surgical mechanical debridement alone of severe furcation involvements usually leads to disease progression. Moreover, it has been observed that the establishment of a correct tooth anatomy that allows optimal plaque control through resective surgical procedures yielded—after 10 years—a 93% survival rate of molars with FI [41].

Taking into account all of the above mentioned aspects, the prognosis of teeth with furcation lesions and, especially, the choice of an appropriate treatment would depend on the characteristics of the lesion, whether it is clinically exposed or not exposed, since according to our understanding and based on previously evaluated literature, in the case of a Grade II–III non-exposed furcation lesion the surgical treatment could be the right approach for a better long term survival rate.

It is important to consider that the present new system measures the horizontal attachment loss alone. Considering previous articles and, to the base of our knowledge and experience, vertical attachment loss in furcation lesion is more difficult to be clinically determined. On the other hand, the morphology of inter-radicular osseous defects is further complicated by the fact that supra-bony and/or infra-bony defects of different morphology may also be associated with furcation involvements [42]. A further aid to their clinical diagnosis is the use of trans-gingival probing or bone sounding [43].

## 5. Conclusions

No classification system can be complete but with its continual use both advantages and disadvantages of each system can be evidenced. This classification attempts to refine the existing drawbacks of the current classifications so that the new system can be applied to a wider variety of cases to provide more accurate characterization of the lesions. This would be of significant aid in communication between clinicians and researchers providing a better understanding of furcation involvements and could be important to predict the prognosis and select correct treatment for each case.

## Figures and Tables

**Table 1:** Review of classifications of furcation involvement.

Authors	Description
Glickman, I. [2]	Grade I: Incipient lesion. Suprabony pocket and slight bone loss in the furcation area. Grade II: Loss of interradicular bone and pocket formation but a portion of the alveolar bone and periodontal ligament remain intact. Grade III: Through-and-through lesion. Grade IV: Through-and-through lesion with gingival recession, leading to a clearly visible furcation area.
Goldman, H.M. [8]	Grade I: Incipient lesion. Grade II: Cul-de-sac lesion. Grade III: Through-and-through lesion.
Staffileno, H.J. [9]	Class I: Furcations with a soft tissue lesion extending to furcal level but with minor degree of osseous destruction. Class II: Furcations with a soft tissue lesion and variable degree of osseous destruction but not a through-and-through communication through the furca. Class II F: Furcations with osseous destruction from facial aspect only. Class II L: Furcations with osseous destruction from lingual aspect only. Class II M: Furcations with osseous destruction from mesial aspect only. Class II D: Furcations with osseous destruction from distal aspect only. Class III: Furcations with osseous destruction with through-and-through communication.
Easley, J.R. et al. [6]	Class I: Incipient involvement, but there is no horizontal component to the furca. Class II: Type 1. Horizontal attachment loss into the furcation. Type 2. Vertical attachment loss into the furcation. Class III: Through-and-through attachment loss into the furcation. Type 1. Horizontal attachment loss into the furcation. Type 2. Vertical attachment loss into the furcation.
Hamp, S.E. et al. [3]	Degree I: Horizontal attachment loss < 3 mm. Degree II: Horizontal attachment loss > 3mm not encompassing the total width of the furcation area. Degree III: Horizontal through-and-through destruction of the periodontal tissue in the furcation area.
Rosemberg, M.M. [10]	Horizontal Degree I: Probing < 4 mm. Degree II: Probing > 4 mm. Degree III: Two or three furcations classified as degree II are found. Vertical Shallow: Slight lateral extension of an interradicular defect, from the center of the trifurcation in a horizontal direction. Deep: Internal furcation involvement but not penetrating the adjacent furcation.
Ramjford, S.P. et al. [11]	Class I: Tissue destruction < 2 mm (1/3 of tooth width) into the furcation. Class II: Tissue destruction > 2 mm (>1/3 of tooth width). Class III: Through-and-through involvement.
Goldman, H.M. et al. [12]	Degree I: Involves furcation entrance. Degree II: Involvement extends under the roof of furcation. Degree III: Through-and-through involvement.
Richietti, P.A. [13]	Class I: 1 mm of horizontal invasion. Class Ia. 1–2 mm of horizontal invasion. Class II: 2–4 mm of horizontal invasion. Class IIa. 4–6 mm of horizontal invasion. Class III: >6 mm of horizontal invasion.
Tal, H. et al. [14]	Furction involvement index (FII) scores: Furcal rating 1: Depth of the furcation is 0 mm. Furcal rating 2: Depth of the furcation is 1–2 mm. Furcal rating 3: Depth of the furcation is 3 mm. Furcal rating 4: Depth of the furcation is 4 mm or more.
Tarnow, D. et al. [4]	For each class of horizontal classification (I–III), a subclass based on the vertical bone resorption was added: Subclass A: 0–3 mm. Subclass B: 4–6 mm. Subclass C: >7 mm.
Eskow, R.N. et al. [15]	Furcation involvement is classified as grade I subclasses A, B, and C (vertical involvement): Subclass A: Vertical destruction > 1/3. Subclass B: Vertical destruction of 2/3. Subclass C: Vertical destruction beyond apical third of interradicular height.
Fedi, P.F. [16]	Glickman + Hamp classifications Grades are the same as Glickman’s classification (I–IV). Grade II is subdivided into degrees I and II. Degree I. Vertical bone loss 1–3 mm. Degree II. Vertical bone loss > 3 mm, but not communicate through-and-through.
Grant, D.A. et al. [17]	Class I: Involvement of the flute only. Class II: Involvement partially under the roof. Class III: Through-and-through loss.
Basaraba, N. [18]	Class I: Initial furcation involvement. Class II: Partial furcation involvement. Class III: Communicating furcation involvement.
Carnevale, G. et al. [19]	Degree I: Horizontal attachment loss < 1/3 Degree II: Horizontal attachment loss > 1/3. Degree III: Horizontal through-and-through destruction.
Hou, G.L. et al. [7]	Classification based on root trunk length and horizontal and vertical bone loss. Types of root trunk: Type A: Furcation involving cervical third of root length. Type B: Furcation involving cervical third and cervical two thirds of root length. Type C: Furcation involving cervical two thirds of root length. Classes of furcation: Class I: Horizontal loss of 3 mm. Class II: Horizontal loss > 3 mm. Class III: Horizontal “ through-and-through” loss. Subclasses by radiographic assessment of the periapical view: Sub-class ‘a’. Suprabony defect. Sub-class ‘b’. Infrabony defect. Classification of furcation: AI, AII, AIII. Type A root trunks with class I, class II and class III furcations. BI, BII, BIII. Type B root trunks with class I, class II and class III furcations. CI, CII, CIII. Type C root trunks with class I, class II and class III furcations.
Nevins, M. et al. [20]	Class I: Incipient or early loss of attachment. Class II: A deeper invasion and loss of attachment that does not extend to a complete invasion. Class III: Complete loss of periodontium extending from buccal to lingual surface. Diagnosed radiographically and clinically.
Glossary of periodontal terms. [1]	Class I: Minimal but notable bone loss in furcation. Class II: Variable degree of bone destruction but not extending completely through furcation. Class III: Bone resorption extending completely through furcation.
Walter, C.et al. [21]	Modification of the Hamp et al. classification (degree II is divided into degrees II and II–III) Degree I: Horizontal attachment loss < 1/3 of the width of the tooth. Degree II: Horizontal loss of support > 3 mm, < 6 mm. Degree II–III: Horizontal loss of support > 6 mm, but not extending completely through furcation. Degree III: Horizontal through-and-through destruction.

**Table 2:** Proposed classification system of furcation involvement.

Type/Grade of Furcation Lesion	Characteristics
**NEI**	The furcation lesion is not clinically exposed. The horizontal attachment loss is 2 mm or less.
**NEII**	The furcation lesion is not clinically exposed. The horizontal attachment loss is 3 mm or more.
**NEIII**	The furcation lesion is not clinically exposed. The horizontal attachment loss is total, with through and through opening of the furcation.
**EI**	The furcation lesion is clinically exposed. The horizontal attachment loss is 2 mm or less.
**EII**	The furcation lesion is clinically exposed. The horizontal attachment loss is 3 mm or more.
**EIII**	The furcation lesion is clinically exposed. The horizontal attachment loss is total, with through and through opening of the furcation.

NE—non exposed; E—exposed.
